# Cephalometric Norms for Mewari Children using Steiner’s Analysis

**DOI:** 10.5005/jp-journals-10005-1161

**Published:** 2012-12-05

**Authors:** Ambika Singh Rathore, Vineet Dhar, Ruchi Arora, Amish Diwanji

**Affiliations:** Assistant Professor, Department of Pediatric and Preventive Dentistry Government Dental College and Hospital, Jaipur, Rajasthan, India e-mail: dr.ambika.rathore@gmail.com; Associate Professor, Department of Pediatric Dentistry, Health Promotion and Policy, University of Maryland Dental School, Baltimore Maryland, USA; Professor and Head, Department of Pedodontics, Darshan Dental College and Hospital, Udaipur, Rajasthan, India; Senior Lecturer, Department of Pedodontics, Faculty of Dental Science, Dharmsinh Desai University, Nadiad, Gujarat, India

**Keywords:** Lateral cephalometric radiograph, Steiner analysis, Mewari children, Cephalometric norms

## Abstract

A thorough background in craniofacial growth and development is necessary for every dentist. An important concept in the study of growth and development is variability. Cephalometrics is an important part of morphological diagnostic procedures to assess craniofacial growth and development. The aim of this study was to obtain cephalometric norms for Mewari children of Rajasthan by Steiner analysis and compare with Caucasian norms. The method involved clinical examination, collection and analysis of 100 lateral cephalometric radiographs of Mewari children (50 males and 50 females, between 11 and 13 years of age). All cephalometric landmarks were located and determined and subsequently tracing was done according to Steiner analysis. The mean value and standard deviation of each measurement were calculated. Statistical comparison was done using Student t-test. The result of this study showed that the Mewari children had retrusion of mandible relative to cranial base, proclined maxillary and mandibular teeth, with greater convexity of face. They also showed anteriorly placed occlusal plane to cranium and Less prominent chin. In conclusion, these ethnic differences should be considered during orthodontic treatment.

**How to cite this article:** Rathore AS, Dhar V, Arora R, Diwanji A. Cephalometric Norms for Mewari Children using Steiner’s Analysis. Int J Clin Pediatr Dent 2012;5(3):173-177.

## INTRODUCTION

A thorough background in craniofacial growth and development is necessary for every dentist. An important concept in the study of growth and development is variability.^1^ Everyone is not alike in the way that they grow; there is always diversity in growth pattern. Rather than categorizing people as normal or abnormal, it is more useful to think in terms of deviations from usual patterns and to express variability quantitatively. Radiographic cephalometrics is a radiographic technique for abstracting the human skull into a geometric scheme.^1^ It is an important part of morphological diagnostic procedures to assess craniofacial growth and development. It allows changes associated with growth to be observed. To diagnose and classify a malocclusion, the measured values of cephalometric parameters are compared with standard values.

Steiner CC (1953)^2^ published a method of interpreting both the hard and soft tissues using cephalometric radiographs. Steiner proposed the appraisal of various parts of the skull separately, namely the skeletal, dental and soft tissue. The skeletal analysis entails relating the upper and lower jaws to the skull and to each other. The dental analysis entails relating the upper and lower incisor teeth to their respective jaws and to each other. And, the soft tissue analysis provides the balance and harmony of the lower facial profile.

The craniofacial features both skeletal as well as dental are either genetically in origin; nutritionally acquired or dietary patterns acquired from parents and are specific to some ethnic, racial, subracial as well as from different community groups. Rajasthan is one of the largest state in India and Mewar is one of the major areas of it. With the increasing number of children of Rajasthan seeking professional treatment for malocclusion, it has become apparent that there is need to determine what constitutes a pleasing or normal face for the children of Rajasthan. A comprehensive and accurate diagnostic assessment of any orthodontic patient involves the comparison of the patient’s cephalometric findings with the norms of his or her ethnic groups or racial groups or subgroups.

## AIMS AND OBJECTIVES

- To determine cephalometric norms for Mewari children (males and females) of Rajasthan between the age group of 11 and 13 years using Steiner’s analysis.
- To compare values obtained for Mewari children using Steiner’s analysis with the values/norms given by Steiner for Caucasian population.

## MATERIALS AND METHODS

This cephalometric radiographic study was carried out in the Department of Pedodontics and Preventive Dentistry, Darshan Dental College and Hospital, Udaipur. Ethical Clearance was taken for the study.

### Source of Data

A total of 100 children, with equal male and female ratio, between the age group of 11 and 13 years were taken for the study from various schools of Mewar region of Rajasthan.

### Method of Collection of Data

Inclusion Criteria

- Subjects with Angle’s class I occlusion
- Normal overjet and overbite
- No crossbite
- Competent lips
- To ascertain the place of origin of the subject, the family linkage of each subject was traced up to three generations
- Subjects with history of systemic disease were excluded from the study
- Consent from the parents of each subject was taken after explaining the nature and purpose of radiograph.

### Radiographic Technique

**Fig. 1:**
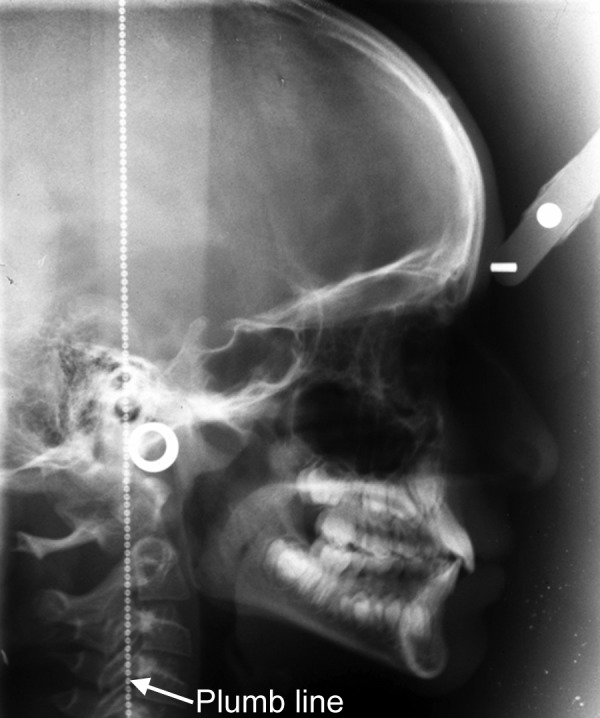
Lateral cephalogram

**Fig. 2:**
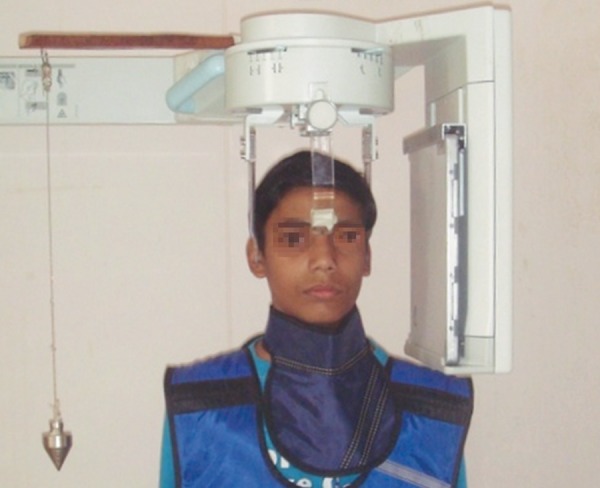
Subject positioning for lateral cephalometric projection

To have standardized cephalometric radiographs, it becomes important that all the radiographs should have similar patient orientation and also same amount of magnification. For this purpose all the lateral cephalometric radiographs were taken from the standardized Ortho Ralix 9200, Gendex OPG machine with a Cephalostat (Dentsply Italia, Italy) on a standard Kodak C-MAT Green sensitive 8 × 10 inch film with an anode- to-midsubject distance of 5 feet. Subjects were asked to look straight in a long mirror which was placed in front of them and plumb line was placed on right side of the subject to obtain natural head position (Fig. 1). Thyroid shield and lead apron was worn by the subject to reduce radiation exposure (Fig. 2). All the films were exposed with 80 KVp, 7.5 mA and an exposure time of 2.5 seconds.

**Fig. 3:**
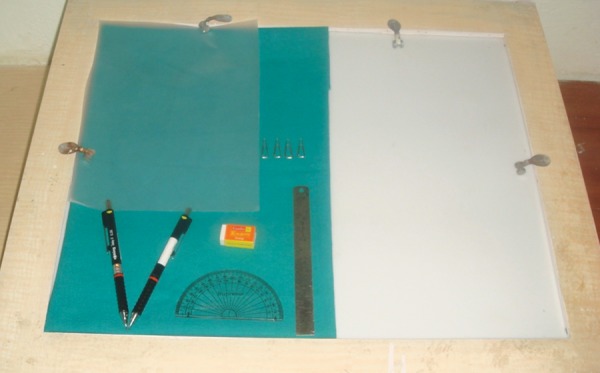
Equipments used for tracing: Lead acetate tracing paper, paper clips, metal scale, erazer, protractor, pencil

**Fig. 4:**
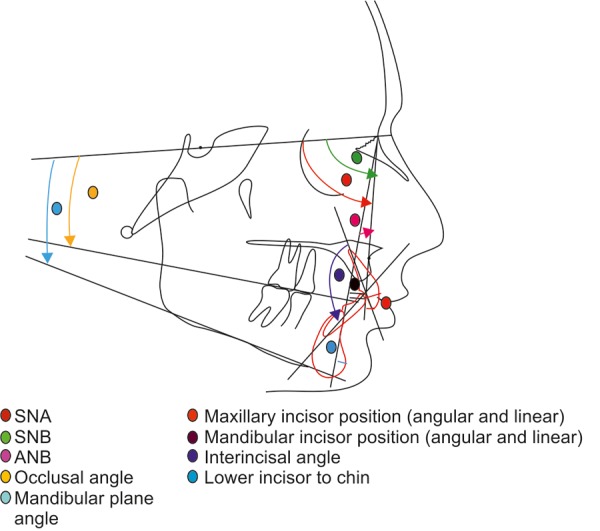
Steiner’s analysis

All the radiographs were traced on a standard matte acetate tracing paper in a random order by a single operator in order to reduce bias (Figs 3 and 4). Each landmark and point was rechecked and then Steiner’s analysis was done.

## RESULTS

The collected data was analyzed statistically using SPSS software (version 10 Inc Chicago, IL). The mean value and standard deviation of each measurement were calculated (Table 1). Student t-test was used to compare measurements of Mewari children with measurements given by Steiner (Table 2).

**Table 1:** **Table 1:** Cephalometric norms for Mewari children using Steiner’s analysis (derived from 100 children)

*Parameters*		*Min*		*Max*		*Mean*		*SD*	
SNA angle		74°		88°		81.06°		2.93°	
SNB angle		70°		86°		77.15°		2.52°	
ANB angle		1°		7°		3.05°		1.38°	
Occlusal angle		14°		24°		19.73°		1.52°	
Mandibular plane angle		24°		33°		30.36°		1.59°	
Maxillary incisor (angular)		14°		34°		23.98°		5.19°	
Maxillary incisor (mm)		0		11		5.11		2.39	
Mandibular incisor (angular)		18°		38°		28.36°		4.18°	
Mandibular incisor (mm)		1		12		5.345		2.07	
Interincisal incisor angle		105°		138°		123.63°		7.19°	
Lower incisor to chin (mm)		– 2		4.5		1.71		1.38	

**Table 2:** **Table 2:** Comparison of measurements between Mewari children and measurements given by Steiner’s analysis

*Parameters*		*t-value*		*p-value*	
SNA angle		– 3.20		≤0.01	
SNB angle		– 4.37		≤0.01	
ANB angle		7.60		≤0.01	
Occlusal angle		37.69		≤0.01	
Mandibular plane angle		– 10.31		≤0.01	
Maxillary incisor (angular)		3.81		≤0.01	
Mandibular incisor (mm)		4.66		≤0.01	
Mandibular incisor (angular)		8.03		≤0.01	
Mandibular incisor (mm)		6.49		≤0.01	
Interincisal angle		– 8.85		≤0.01	
Lower incisor to chin (mm)		– 16.54		≤0.01	

Student t-test (p-value **≤** 0.01, significant, nonsignificant)

## DISCUSSION

This study was carried out to determine cephalometric norms for Mewari children of Rajasthan between the age group of 11 and 13 years. The ranges of most of the dimensions of the present study were significantly different than those obtained by Steiner, although all selected individuals had a pleasant appearance and good facial harmony.

*SNA angle:* The mean value of SNA angle in the present study was slightly less in Mewari children, i.e. (81.06° ± 2.93°) than those presented by Steiner (82 ± 2°),^2^ indicating maxillary retrusion relative to cranial base for Mewari children as compare to those given by Steiner. The measurement of SNA angle for Mewari children (81.06 ± 2.93°) is in agreement with the study done by Chandranee^3^ (1982) on North Indian children (SNA-81.6°).

*SNB angle:* The mean value of SNB angle in the present study was significantly less in Mewari children (77.15° ± 2.52°) than those presented by Steiner (80 ± 2°),^2^ indicating mandibular retrusion relative to cranial base. The SNB angle found in present study is in agreement with the studies done by Chandranee^3^ (1982), (SNB-78.5°) and Kharbanda OP^4^ et al, (1989), (SNB-78.52°) on North Indian and Aryo- Dravidians respectively.

*ANB angle:* The mean value of ANB angles for Mewari (3.05 ± 1.38°) children was slightly more than those presented by Steiner^2^ (2°), indicating greater convexity. This is in agreement with the study done by Chandranee^3^ (1982) on North Indian children (ANB-3.12°), Anuradha^5^ et al (1990) on North Indian preschool children (4.95°) and Abraham KK^6^ in (2001) on children of South Kanara (ANB- 3.2°) significant difference of ANB angle in all these studies was found when compared with those presented by Steiner.

*Occlusal plane angle:* In the present study, the mean of occlusal plane angle for Mewari (19.73° ± 1.52°) children showed a significant difference than measurements given by Steiner (14°).^2^ This indicates more anteriorly placed occlusal plane as compared to those given by Steiner. This is in agreement with the study done by Anuradha^5^ et al (1990) on North Indian preschool children (occlusal angle— 21.7°) which is greater than those presented by Steiner.

Mandibular plane angle suggests growth patterns in individuals, the mean values of this angle for Mewari (30.36 ± 1.59°) children was slightly less than those presented by Steiner^2^ (32°). This finding is in agreement with the study done by Joshi^7^ (1975) on dentofacial patterns of Gurkhas (mandibular plane angle—29.7°) and Kannappan JG^8^ et al (1976) on Madras population (mandibular plane angle–31.0°), where mandibular plane angle was lesser than the value given by Steiner.

Maxillary incisor position represents the relative location and axial inclination of the upper incisors.

The upper incisor to N-A reading in degrees indicates the relative angular relationship of the upper incisor teeth to N-A line, the mean value of maxillary incisor position in degrees in present study for Mewari (23.98 ± 5.19°) children is significantly higher than those presented by Steiner indicating more labial inclination of maxillary teeth in Mewari children.

The upper incisors to N-A reading in millimeters provides information on the relative forward or backward positioning of the incisor teeth to N-A line, the mean value in present study for Mewari (5.11 ± 2.39 mm) children is significantly higher than those presented by Steiner indicating more forward positioning of maxillary teeth in Mewari and children.

This finding is in agreement with the study done by Valiathan A^9^ (1975) on Indian population in which she had concluded that the teeth of people from India were more labially placed. This finding is also in agreement with the study done by Kannappan JG^8^ et al (1976) on Madras population (Angular measurement—23.5°, linear measurement—4.2 mm) and also study done by Chandranee^3^ (1982) on North Indian children (angular measurement- 24.9°, linear measurement-4.9 mm) where both the angular and linear measurements were more as compared to those given by Steiner.

Mandibular incisor position represents the relative anteroposterior location and angulation of the lower incisor teeth.

The lower incisor to N-B line in degrees indicates relative angular relation. The mean value of mandibular incisor position in degrees in present study for Mewari (28.36 ± 4.18°) children is significantly higher than those presented by Steiner indicating more labial inclination of mandibular teeth in Mewari children.

The lower incisors to N-B reading in millimeters provides information on the relative forward or backward positioning of the incisor teeth to N-B line, the mean values in present study for Mewari (5.34 ± 2.07 mm) children is significantly higher than those presented by Steiner, indicating more forward positioning of mandibular teeth in Mewari children.

This finding is in agreement with the study done by Valiathan A^9^ (1975), Valiathan A^10^ (1976) on Indian population in which she had concluded that the incisor teeth of people from India were more labially placed. This finding is also in agreement with the study done by Kannappan JG^8^ et al (1976) on Madras population (angular measurement- 26°, linear measurement—5.2 mm) and also in a study done by Chandranee^3^ (1982) on North Indian children (angular measurement—27.8° and linear measurement 6 mm) where both the angular and linear measurements were more as compared to that given by Steiner.

Interincisal angle relates the relative position of the upper incisor to that of the lower incisors. The mean value of interincisal angle in present study for Mewari (123.63 ± 7.19°) children is significantly lower than those given by Steiner, indicating proclined maxillary and mandibular teeth.

This finding is in agreement with the study done by Nanda R^11^ et al (1969) on North Indians in which they had reported acute interincisal angle suggesting more vertical incisors in North Indians, Valiathan A^9^ (1975) on Indian population (Interincisal angle-119°) in which she had concluded that the incisor teeth of people from India were more labially placed.

This finding is also in agreement with the study done by Elbe P^12^ et al (2000) in which it was found that interincisal angle was less for North Indians as compared to Caucasians and was concluded that North Indians have more proclined lower incisors as compared to Caucasians.

Lower incisor to chin indicates the prominence of chin when compared with lower incisors. The mean value of lower incisor to chin in present study for Mewari (1.71 ± 1.38 mm) children is significantly lesser than those presented by Steiner (4 mm), indicating less prominence of chin to lower incisors for Mewari children as compared to those given by Steiner.

## CONCLUSION

- Mewari children showed retrusion of mandible relative to cranial base
- Greater convexity of face was found for Mewari children
- Mewari children showed anteriorly placed occlusal plane to cranium
- Mewari children showed proclined maxillary and mandibular teeth
- Less prominent chin was found for Mewari children.
